# The complete chloroplast genome of *Primulina* and two novel strategies for development of high polymorphic loci for population genetic and phylogenetic studies

**DOI:** 10.1186/s12862-017-1067-z

**Published:** 2017-11-07

**Authors:** Chao Feng, Meizhen Xu, Chen Feng, Eric J. B. von Wettberg, Ming Kang

**Affiliations:** 1 Key Laboratory of Plant Resources Conservation and Sustainable Utilization, South China Botanical Garden, Chinese Academy of Sciences, 723 Xingke Road, Guangzhou, 510650 China; 2 University of Chinese Academy of Sciences, Beijing, 100049 China; 3 Department of Plant and Soil Sciences, University of Vermont, Burlington, VT 05405 USA

**Keywords:** *Primulina*, Next-generation sequencing, RAD-Seq, Chloroplast assembly, High-variation regions, Sub-super-marker

## Abstract

**Background:**

*Primulina* Hance is an emerging model for studying evolutionary divergence, adaptation and speciation of the karst flora. However, phylogenetic relationships within the genus have not been resolved due to low variation detected in the cpDNA regions. Chloroplast genomes can provide important information for phylogenetic and population genetic studies. Recent advances in next-generation sequencing (NGS) techniques greatly facilitate sequencing whole chloroplast genomes for multiple individuals. Consequently, novel strategies for development of highly polymorphic loci for population genetic and phylogenetic studies based on NGS data are needed.

**Methods:**

For development of high polymorphic loci for population genetic and phylogenetic studies, two novel strategies are proposed here. The first protocol develops lineage-specific highly variable markers from the true high variation regions (Con_Seas) across whole cp genomes, instead of traditional noncoding regions. The pipeline has been integrated into a single perl script, and named "Con_Sea_Identification_and_PIC_Calculation". The second method assembles chloroplast fragments (poTs) and sub-super-marker (CpContigs) through our "SACRing" pipeline. This approach can fundamentally alter the strategies used in phylogenetic and population genetic studies based on cp markers, facilitating a transition from traditional Sanger sequencing to RAD-Seq. Both of these scripts are available at https://github.com/scbgfengchao/.

**Results:**

Three complete *Primulina* chloroplast genomes were assembled from genome survey data, and then two novel strategies were developed to yield highly polymorphic markers. For experimental evaluation of the first protocol, a set of *Primulina* species were used for PCR amplification. The results showed that these newly developed markers are more variable than traditional ones, and seem to be a better choice for phylogenetic and population studies in *Primulin* *a*. The second method was also successfully applied in population genetic studies of 21 individuals from three natural populations of *Primulina*.

**Conclusions:**

These two novel strategies may provide a pathway for similar research in other non-model species. The newly developed high polymorphic loci in this study will promote further the phylogenetic and population genetic studies in *Primulina* and other genera of the family Gesneriaceae.

**Electronic supplementary material:**

The online version of this article (10.1186/s12862-017-1067-z) contains supplementary material, which is available to authorized users.

## Background

Chloroplast sequences are important molecular tools for studies of plant phylogeny, phylogeography and population genetics [1–4]. Traditionally, selected cpDNA regions have been chosen for analysis, mostly based on their conservation and efficacy in related taxa. By comparing chloroplast genomes of 13 angiosperm lineages, Shaw et al., [5, 6] identified a set of 34 non-coding regions that ranked highest in their potentially informative characters (PIC), an index which is counted by the sum of nucleotide substitutions, indel and inversions between each of two ingroup species and between an ingroup species and an outgroup species. This set of most variable non-coding regions is consequently widely used in plant evolutionary biology and systematics studies. However, recent comparative plastid genomic studies reveal considerable variation and surprisingly little (*c*. 12–25%) overlap in the most variable non-coding regions among different lineages [3, 7–10]. Furthermore, around one third of universal barcoding primers were unlikely to work across all the angiosperms [11]. These findings imply that lineage-specific screening is needed for the identification of the most highly variable markers in different clades.

On the other hand, recent advances in next-generation sequencing (NGS) techniques greatly facilitate the sequencing of whole cp genomes for multiple individuals at relatively low cost [12–14]. However, molecular phylogenetic studies of whole chloroplast genome sequences are yet not practical for large clades with hundreds of species, due in part to insufficient capacity to assemble and analyze such large amounts of NGS data. Although several technical innovations have been proposed for cpDNA assembly based on NGS data [15–17], novel strategies aimed at more time-saving, labor-saving and cost-saving are desirable. Paired-end RAD-Seq (restriction-site associated DNA sequencing) [18] could prove to be an efficient tools for obtaining large numbers of partial chloroplast genomes. Owing to the partial cpDNA sequences that RAD-Seq may provide, it can facilitate chloroplast-based phylogenetic reconstruction with high resolution [19–21]. However, to date, methods specific for chloroplast sub-assembly from paired-end RAD-Seq have not been developed.

*Primulina* Hance is a large genus of the Old World Gesneriaceae with *c.* 170 species that are widely distributed throughout the limestone karst regions of southern China and Southeast Asia, one of the world’s biodiversity hotspots [22]. This genus is uniquely suited for studying evolutionary divergence, adaptation and speciation of the karst flora, due to its high species richness and endemism and high degree of habitat specialization [23, 24]. To date, the nuclear ribosomal internal transcribed spacer (ITS) and the plastid non-coding regions *trnL-trnF*, *rpl32-trnL* and *atpB-rpl32* have been used to reconstruct the relationships of *Primulina* [25–27]. These studies provide a phylogenetic framework for the genus. Kang et al., [27] sampled 104 taxa to reconstruct the largest phylogeny of *Primulina* so far, in which four major clades were resolved. However, phylogenetic relationships among many taxa have not been resolved due to low variation in these cpDNA regions. Recently, genomic resources have been developed for several species by using RNA-Seq [23]. Besides the nuclear genome, the complete chloroplast genome of *Primulina* can provide important insights into phylogenetic relationship and evolutionary history of this genus.

Here we present three complete chloroplast genomes of *Primulina*, and two novel strategies to develop highly polymorphic cp markers. The first strategy develops cp primers from all the highly variable regions (called Con_Seas) across whole cp genome, instead of just traditional noncoding regions. The pipeline has been integrated into a single perl script, and named “Con_Sea_Identification_and_PIC_Calculation”. The second strategy uses RAD-Seq to directly assemble cp fragments (poTs) and sub-super-marker (CpContigs). This second approach represents a fundamental shift away from cp-primer based Sanger sequencing because it creates a reduced cp genome, which could be used for population genetic analysis as well as phylogenetics. The core pipeline, SACRing, was written in bash, combining several popular software tools and our in-house perl scripts. Both of these scripts are available at https://github.com/scbgfengchao/, and will be continual improved and updated. Here the performance of these methods was evaluated in an experimental dataset.

## Methods

### Plant material and DNA extraction

Three individuals each from *P. linearifolia* (population code, GXNN01), *P. huaijiensis* (GDHJ02)*, P. eburnea* (WHY01) were used for genome survey sequencing and completed chloroplast genome assembly. Forty-nine individuals from 44 *Primulina* species (Additional file 1: Table S1) were used to evaluate polymorphism of cp markers developed in this study. In addition, restriction-site associated DNA sequences (RAD-Seq) of 21 individuals from three natural populations, seven each from *P. eburnea* (CZYX01), *P. yongxingensis* (CZYX02) and *P. juliae* (CZYX03), were used for partial cp genome assembly. All leaf samples were frozen in liquid nitrogen and stored at −80 °C. Total DNA was extracted from the leaves using a modified CTAB method [28] and treated with RNase (TransGen, China).

### Library construction, Illumina sequencing and quality control

For genome survey sequencing, DNA from 3 samples (GXNN01, WHY01 and GDHJ02) was broken into the short fragments with the length of 180 bp, 230 bp and 230 bp, respectively, using focused-ultrasonicators (Covaris, USA). Two libraries were built for each sample, and further sequenced by paired-end sequencing technology of Illumina HiSeq 2000™. For RAD-Seq of the three populations, DNA was first treated with restriction enzyme *EcoR I* (Takara, China), and then several standard steps were performed, from the addition of sequencing adapters, interruption of enzyme digestion products, to break into smaller random pieces, and repairing the end based on existing protocols [18]. Finally, fragments with lengths ranging from 200 to 800 bp were separated on an agarose gel and selected for PCR amplification as sequencing templates. These libraries were sequenced by PE 100 model on Illumina HiSeq 2000™. The programs of library construction and Illumina sequencing was performed by staff of Novogene Bioinformatics Institute (Beijing, China).

The raw reads were first filtered by removing the adapter sequences and low quality sequences using Software FASTX_Toolkit (http://hannonlab.cshl.edu/fastx_toolkit/index.html) and our in-house perl scripts. Only the reads with a Q20 percentage (i.e., the percentage of sequences with sequencing error rate lower than 1%) over 90% and N percentage (i.e., the percentage of nucleotides in read which could not be sequenced) less than 5%, were marked as clean data and used for further analysis. The sub-routine above was integrate into the pipeline, and named QC_pe (Additional file 2: File S1), which is available at https://github.com/scbgfengchao/.

### Complete chloroplast assembly from genome survey data

*P. linearifolia* was used for the first pass of genome survey sequencing and complete chloroplast genome assembly. Briefly, we used the software of Bowtie2 (version 2.2.5) [29] with the parameter "-I 150 -X 1000 --no-mixed --no-discordant". The cp data of *P. linearifolia* was isolated by mapping it to the cp genome of *Boea hygrometrica* (GenBank accession id: NC_016468), a closely related species belonging to the Gesneriaceae family with a publically available cp genome sequences [30]. Furthermore, the cp genome of *P. linearifolia* was sub-assembled using the Velvet software (version 1.2.10) [31], with parameter hash_length (kmer_length) setting from 29 to 99. The sub-assembly with longest contig N50 was selected and further scaffolding based on the original cp data, using SSPACE software (version 3.0) [32] with default parameter (−m 32 -o 20 -r 0.9). After that, each scaffold was located against the cp genome of *Boea hygrometrica* using blat software [33]. The gaps between scaffolds were closed by PCR amplifications and Sanger sequencing. Meanwhile, Sanger sequences were used for nucleotide confirmation, especially at LSC/IR/SSC boundaries.

The complete cp genomes of *P. eburnea* and *P. huaijiensis* were obtained as described above in turn. The difference is that the cp genome of *P. linearifolia*, instead of *B. hygrometrica*, was used as reference genome to isolate the cp data, and the upper limit of hash_length was set as 125 in Velvet software. Finally, in order to verify the accuracy of the assembled cp genomes, the cp data of the three *Primulina* species was mapped back to their respective cp genome by using the program SAMtools (version 0.1.19) [34]. The mapping results were displayed with IGV software (version 2.3.57) [35].

### Genome annotation and sequence alignment

The cp genomes of *B. hygrometrica* and the three *Primulina* species were annotated using the online program DOGMA (http://dogma.ccbb.utexas.edu/) [36], and modified by detailed manual corrections. The tRNA boundaries and splice sites were modified by using tRNAscan-SE software (version 1.3.1) [37]. The annotated cp genome maps were drawn by using the online OrganellarGenomeDRAW tool (OGDRAW) [38] and local software Circos (version 0.67) [39].

A consensus sequence was obtained by clustalW alignment of the 4 cp genomes (*B. hygrometrica* and three *Primulina* species) with manual corrections, especially in the area of exon boundaries. Meanwhile, the correspondence between each cp genome and consensus sequence was built using our in-house perl script (Additional file 3: File S2; also available at https://github.com/scbgfengchao/). Furthermore, the orthologous coding exons (defined as syntenic coding loci) and orthologous noncoding intergenic regions/introns (defined as syntenic noncoding loci) among 4 cp genomes were classified and identified.

### Determination of Con_Sea regions and calculation of polymorphic index

Based on the alignment results, conserved sites among the 4 cp genomes were labeled. “Con_Islands” (defined as regions containing over 50 continuous conserved sites in the cross-genus consensus genome sequences) were first identified, while the regions between two adjacent Con_Islands were named “Con_Seas”.

The potentially informative characters (PICs), an index counted by the sum of SNP and Indel between two chloroplast genomes, was used to evaluate the polymorphism in each Con_Sea region. The pipeline above has been integrated into a single perl script, and named “Con_Sea_Identification_and_PIC_Calculation” (https://github.com/scbgfengchao/, Additional file 4: File S3). The parameter “minimum length of Con_Island” (50 in this study) can be varied. There is no other software dependency for this script, so it targeted toward researchers without bioinformatics background. PIC at each Con_Sea was divided into intrageneric and intergeneric levels. Intrageneric PIC was calculated from the average PIC values of pairwise of 3 *Primulina* species, while intergeneric PIC were equal to the mean of PIC value of 3 groups between each *Primulina* species and *B. hygrometrica*.

In addition, the intra-population (CZYX01, CZYX02 and CZYX03) PIC values were calculated based on the average PIC of 21 pairs from 7 individuals of respective populations, respectively, while the PIC information for the inter-population pairs were counted by the mean of 49 pairs. These calculations were performed with our in-house perl script “PIC_calculation” (Additional file 5: File S4), and it’s also available at https://github.com/scbgfengchao/.

### Determination of high variation regions and development of chloroplast markers

The intrageneric and intergeneric polymorphisms (PICs) were analyzed using three different methods of dividing genomic regions. The first method is a sliding window analysis along the consensus sequence, setting the window and step sizes as 100 and 25 bp, respectively, then PIC in each window was plotted with the software Circos (version 0.67) [39]. The second and third method is based on gene regions (syntenic coding regions and syntenic noncoding regions) and variable regions (Con_Seas), respectively. The method based on noncoding regions is a traditional strategy to choose highly polymorphic regions, while the third one is a novel strategy first proposed here, and it is a more effective and directed method to screen lineage-specific high variation regions. The PIC in each region, including syntenic noncoding regions and Con_Seas, was displayed with OriginLab Origin (version 8.0) (Microcal Software INc., Northampton, MA, USA). It was worth noting that the length of these regions varies greatly. In general, Sanger sequencing has been widely used to obtain more sequence data from more species or individuals based on polymorphism chloroplast primers, which were developed from high variation regions. Reserving around 100 bp for primer designing, 700 and 1500 bp are effective lengths of single and two-directional Sanger sequencing reactions, respectively. So in this study, these two lengths are considered as key bounds to evaluate fairly the polymorphic degree of regions with different length.

In order to evaluate the efficiency of the identified high polymorphism regions, eight new genus-specific cp markers, developed from high variation regions based on the Con_Seas method, were tested in the 49 individuals from 44 *Primulina* species. For comparison, these individuals were also amplified with four traditional cp markers (*atpB-rbcL*, *rpl32-trnL*, *trnL-trnF* and *trnS-trnG*), which were selected from 19 traditional universal cp primers that have been used in our previous phylogenetic work [27]. It is worth noting that some new cp primers were designed from slighter lower variation regions, in order to provide a more comprehensive comparison among markers.

### Sub-assembly of chloroplast from PE RAD-Seq (SACRing)

**Fig. 1:**
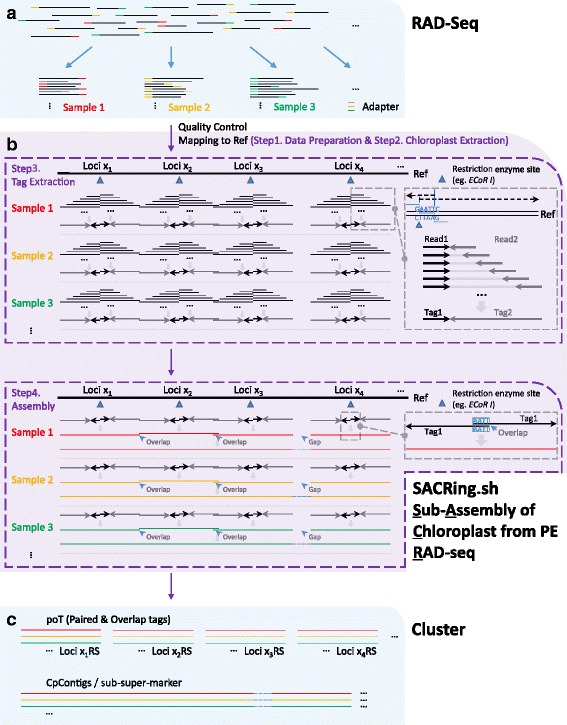
Schematic of sub-assembly of chloroplast genome from PE RAD-seq. **a** Illustration of RAD-seq method. **b** Pipeline for sub-assembly of cpDNA from PE RAD-seq. This pipeline, SACRing, was divided into 4 steps, and step 3 and 4 were highlighted in top and bottom boxes with a purple dashed line, respectively. The detail of assembly was enlarged with the boxes with a grey dashed line. **c** Illustration of cluster analysis based on the result of SACRing

A novel pipeline, SACRing, was performed for sub-assembly of chloroplast sequences from PE RAD-Seq. This pipeline (Additional file 6: File S5) was written in Bash, combing several in-house perl scripts with mainstream software, such as Bowtie (version 1.1.2) [40], Bowtie2 (version 2.2.5) [29], Stacks (version 1.40) [41], CAP3 [42] and blat [33]. The pipeline is publically available at https://github.com/scbgfengchao/, together with the usage, examples, notes and description of output files, with the schematic shown in Fig. 1. In this study, for evaluating this pipeline, RAD-Seq data of 21 individuals from three *Primulina* populations were used for sub-assembly of cp genome, and the cp genome of *P. eburnea* was set as the reference.

First, paired-end RAD-Seq data was separated in different samples according to adapters (Fig. 1a). Following quality control (Additional file 7: Table S5, https://github.com/scbgfengchao/) and previous analysis (Step 1 in SACRing), the cp-related data was extracted from PE RAD-Seq based on mapping results (Step 2 in SACRing). And then, Step 3, the core step, was used for basic clustering and assembly. Hundreds of thousands of Read1 were mapped back to the specific regions of Ref and clustered into several Tag1s. While hundreds or thousands of Read2, which belonged to the same Tag1, were further assembled into a longer Tag2. This step is the most rate-limiting one, so multi-core CPU scripts were designed to linearly shorten the run time. Step 4 was used to constantly and continually assemble for a better result. Tag1s at the same position were mixed into one sequence with the name of paired tags (pTs). Meanwhile, Tag1 and its paired Tag2 were assembled in a contig, and named overlap tags (oTs). If both Tag1s of pT has an overlap with their paired Tag2s, a longer contig was generated, and defined as poTs, i.e., short for paired & overlap tags. In addition, several longer contigs (defined as CpContigs) were assembled according to overlap information. And a scaffold (sub-super-marker) was further generated according to the position information, replacing the unknown bases with ‘-’ (Fig. 1b).

### Population genetic analysis of chloroplast fragments

Based on the SACRing pipeline, poTs and CpContigs were obtained independently from RAD-Seq data from 21 individuals. For poTs, the most conserved ones, appearing in all the individuals, were selected, and further used for isolation of consistent sequences. Moreover, using CAP3 [42], these sequences were assembled into cp fragments (Fig. 1c) and shown with Circos (version 0.67) [39]. These cp fragments were then concatenated for population genetic analysis. The best substitution model was determined using software jModelTest (version 2.1.7) [43], and then the Bayesian phylogenetic tree was generated with MrBayes (version 3.2.6) [44] under the model of “GTR + I”, while Maximum Likelihood phylogenetic tree was performed using RAxML (version 8.2.9) [45] with 1000 replicates under the model of “GTR + I + G”, for model “GTR + I + G” is the second best model for cp fragments (poTs), and model “GTR + I” is not supported in RAxML software. The basic indexes of population genetics, such as nucleotide diversity (π), theta (θ) and the average nucleotide diversity between populations (*F*_ST_), were calculated by DnaSP (version 5.10.1) [46].

Meanwhile, conserved CpContigs of these individuals were also obtained and analyzed as a poTs dataset. In this dataset, “GTR + I + G” was the best substitution model for the construction of a Bayesian phylogenetic tree and a Maximum Likelihood phylogenetic tree.

## Results

### Complete chloroplast genome assembly and genomic organization

**Fig. 2:**
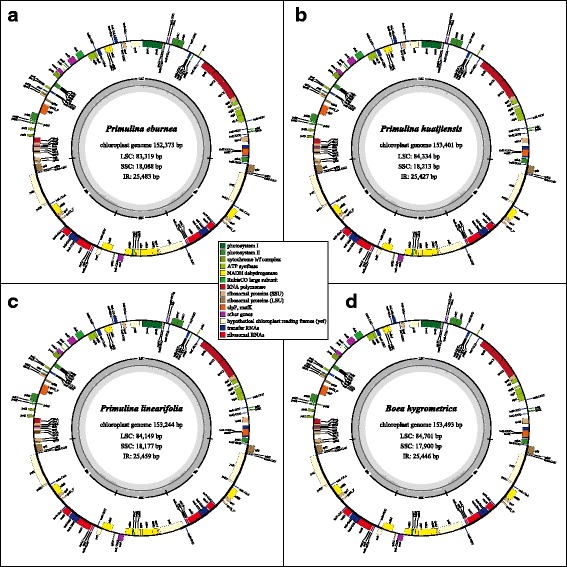
Circular map of chloroplast genomes of three *Primulina* species and *Boea hygrometrica.* Genes shown inside and outside of the outer circle are transcribed clockwise and counterclockwise, respectively. Genes belonging to different groups are marked with different color. The distribution of GC content was shown in the inner circle. Circular map of cp genomes of *P. eburnea* (**a**), *P. huaijiensis* (**b**), *P. linearifolia* (**c**) and *Boea hygrometrica* (**d**)

Using high-throughput genome survey sequencing, we obtained very deep sequence coverage for *P. eburnea* (population code, WHY01), *P. huaijiensis* (GDHJ02) and *P. linearifolia* (GXNN01), ranging from 3.1 to 4.6 G, with Q30 over 95% in all three species (Table 1). Then, three complete *Primulina* cp genomes were assembled, with the average coverage between 20,000 and 30,000 x, and the minimum of 2000 x (Table 1 and Additional file 8: Fig. S1). No SNPs or Indels were identified when mapping the sequencing data back to their respective cp genomes, respectively, indicating that the assembled cp genomes are accurate and high-quality. The full length cp genome of *P. huaijiensis* (153,401 bp) is longest and has the longest SC regions (but shortest IR regions), followed with *P. linearifolia* (153,244 bp) and *P. eburnea* (152,373 bp, as well as shortest SC regions, but longest IR regions) (Fig. 2).

**Table 1:** The basic information of genome survey data of the three *Primulina* species related to chloroplast genomes

Species	Population code	Reads No. (M)^a^	Throughput(G)^b^	Quality Q30^c^	Cp Size (bp)	Coverage
*P. eburnea*	WHY01	12.5	3.14	97.2; 96.9	152,373	20,585
*P. huaijiensis*	GDHJ02	18.5	4.63	97.8; 97.5	153,401	30,191
*P. linearifolia*	GXNN01	21.1	4.22	95.4; 95.4	153,493	27,517

^a^Reads No. was counted based on the Reads which was used in assembly of cp genomes, instead of whole genome survey sequencing data

^b^Throughput = Read No. x read length

^c^Quality Q30 was counted by Read1 and Read2 respectively

The number and order of predicted functional genes are perfectly consistent among cp genomes of *Boea hygrometrica* and the three *Primulina* species (Fig. 2; Additional file 9: File S7 and Additional file 10: Table S2). The cp genome encodes 132 functional genes, with 18 duplicated in the IR regions. Meanwhile, 88 protein coding, 36 tRNA and 8 rRNA genes were identified. Nearly 12.9% of function genes are intron-containing ones, including 10 protein coding genes and 7 tRNA ones, while *clpP* and *ycf3* genes contain two introns (Additional file 10: Table S2). Furthermore, 253 syntenic loci were identified, including 129 syntenic coding loci and 124 syntenic noncoding loci (Additional file 10: Table S2 and Additional file 11: Table S3).

### Sequence divergence and consistency analysis

**Fig. 3:**
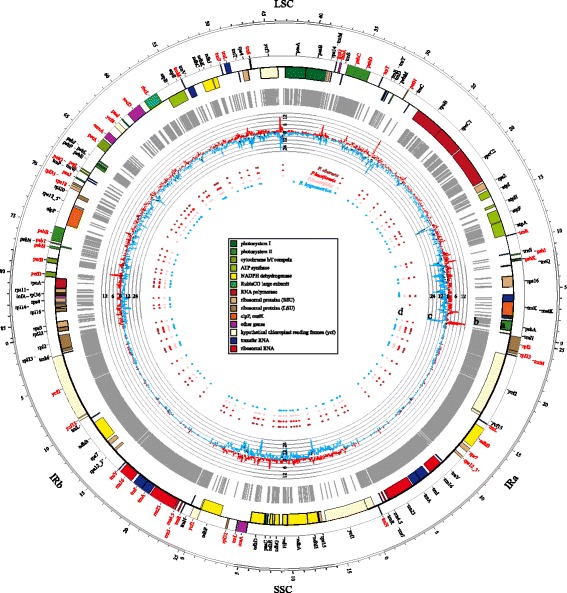
Circular map of cross-genus consensus chloroplast genome sequences of three *Primulina* species and *Boea hygrometrica.* The outermost circle is positions (in Kb) of consensus cp genome sequences. **a** Annotation of consensus cp genome sequences. Genes shown inside and outside of the circle are transcribed clockwise and counterclockwise, respectively, and their gene names are marked as black and red, respectively. Genes belonging to different groups are marked with different color, with the bar shown in the center. **b** Distribution of conserved regions of the four cp genomes. The grey columns represent the Con_Islands, which were defined as the regions containing over 50 continuous conserved sites in cross-genus consensus cp genome sequences. **c** Distribution of intrageneric and intergeneric polymorphism. The red and blue lines represent average intrageneric and intergeneric PICs in a 100 bp windows with a step of 25 bp, respectively. The PICs were counted by the sum of SNP and Indel between two cp genomes. **d** Distribution of restriction enzyme site of *EcoRI*. The outer to inner circles represent the distribution of *EcoR I* in *P. eburnea, P. huaijiensis, P. linearifolia* and *B. hygrometrica* in turns

After alignment and manual correction of the four cp genomes, a cross-genus consensus cp genome sequence with a length of 155,906 bp was obtained, containing a LSC of 86,371 bp, a SSC of 18,455 bp and two IR copies of 25,540 bp (Fig. 3a). Among 155,906 nucleotides, 144,202 (92.5%) are conserved among the four cp genomes, while 151,691 nucleotide (97.3%) are conserved among the three *Primulina* cp genomes (Additional file 12: File S8). The percentage of conserved sites is significantly higher in IR regions (98.5% and 99.4% in the four cp genomes and the three *Primulina* ones, respectively) than SC regions (89.5% and 96.3%), which indicates that intergeneric variation between *Primulina* and *Boea* is mainly ascribed to SC regions.

Furthermore, a total of 622 Con_Islands (the regions containing over 50 continuous conserved sites in a cross-genus consensus cp genome sequence) were identified. The longest one is encoding for partial rrn23 gene in IR regions, with the length of 1145 bp (Fig. 3b). Meanwhile, 622 Con_Seas (the regions between two adjacent Con_Islands) were generated, with the longest one of 1409 bp, located at LSC: 62,228–63,636 bp. 181 Con_Seas have a length of just 1 bp (Fig. 3b and Additional file 13: Table S4). In general, coding regions tend to have longer Con_Islands. Nevertheless, several large Con_Islands were detected in intergenic regions/introns, and partial exons were variant (Fig. 3b).

### Determination of high variation regions

The sliding window analysis revealed that the average intrageneric and intergeneric PICs of all the 6234 windows is 0.82 and 3.51 per 100 bp, respectively. The intrageneric and intergeneric PICs of all the windows have a weak positive correlation (*r* = 0.58; *p* < 0.0001; Additional file 14: Table S5). A total of 18 sliding windows from five regions have intrageneric PIC higher than 9. All of these five regions are located in LSC regions, and the windows with highest PIC (PIC = 19) is around 29 kb in LSC (Fig. 3c and Additional file 14: Table S5). For intergeneric polymorphism, 24 sliding windows from 8 regions have a PIC over 24, with the top PIC of 34.33, located at the position of 15 kb of LSC. Three high intergeneric regions overlap with high intrageneric regions, appearing at the regions near 0, 9 and 44 kb of LSC, respectively (Fig. 3c and Additional file 14: Table S5).

**Fig. 4:**
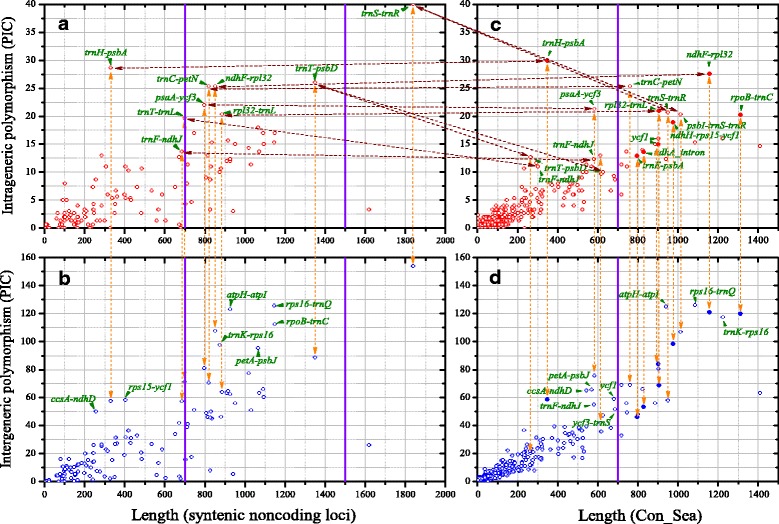
Intrageneric and intergeneric polymorphism of chloroplast variation regions of three *Primulina* species and *Boea hygrometrica*. Intrageneric (**a**) and intergeneric (**b**) polymorphism (PICs) of noncoding regions of cp genomes. Intrageneric (**c**) and intergeneric (**d**) polymorphism (PICs) of Con_Sea regions of cp genomes. PICs were counted by the sum of SNP and Indel between two cp genomes. Con_Sea is a region between two adjacent Con_Islands, which is defined as the regions containing over 50 continuous conserved sites in cross-genus consensus genome sequences. Eight regions used for experimental evaluation were signed as filled circles, while others were empty circles. The name of the regions with high variation or used for experimental evaluation was marked along the circles. It was noting that the name of these regions in part C and D was shown as the name of noncoding regions overlapped with corresponding Con_Seas, instead of original ID of Con_Seas. The yellow lines link the same regions, while the red lines link the corresponding regions between noncoding regions and Con_Seas

The PICs of 129 syntenic coding loci were summarized in Additional file 11: Table S3. Almost all the genes have relatively low intrageneric and intergeneric PIC, with average value of 0.46 and 2.11 per 100 bp, respectively, therefore they are significantly and strongly correlated (*r* = 0.99, *p* < 0.0001). Nevertheless, an exception was discovered in the longest *ycf1* gene (5.5 kb), which has an intrageneric and intrageneric PIC value of 76.33 and 402.67, respectively. This region has the highest polymorphism per 100 bp. While for the 124 syntenic noncoding loci, the average intrageneric and intergeneric polymorphism are 1.30 and 5.52 per 100 bp, respectively, and they are also highly correlated (*r* = 0.88, *p* < 0.0001) (Additional file 11: Table S3). In detail, *trnS-trnR* has both the highest intrageneric and intergeneric PICs, however, its length is much longer than two-directional Sanger sequencing. *TrnH-psbA* has the second highest intrageneric PIC, but relative lower intergeneric PIC. Another seven regions (*trnT-trnL*, *trnF-ndhJ*, *trnT-psbD*, *trnC-petN*, *ndhF-rpl32*, *psaA-ycf3* and *rpl32-trnL*) also have relative high intrageneric PICs (Fig. 4a and Additional file 11: Table S3). For intergeneric PICs, *rps16-trnQ*, *atpH-atpI* and *rpoB-trnC* are listed as the top 2–4 highest; however, their intrageneric ones are relative lower. Similar loci were widely observed, such as *trnK-rps16*, *petA-psbJ*, *rps15-ycf1* and *ccsA-ndhD* (Fig. 4b and Additional file 11: Table S3).

The high variation regions identified based on continuous conserved sites (the third region division method) were summarized in Additional file 13: Table S4. The average intrageneric and intergeneric PICs of 622 Con_Seas are 1.75 and 7.51 per 100 bp, which are significantly higher than the polymorphism of noncoding regions (1.30 and 5.52), respectively. And the correlation coefficient (*r* = 0.90, *p* < 0.0001) is also slightly higher than that of noncoding regions (r = 0.88) (Additional file 13: Table S4). The highest intrageneric polymorphism region is Con_Sea_1, overlapping with *trnH-psbA*. It is a slightly higher PIC (30) than *trnH-psbA* (28.67), because partial *psbA* mutational sites was added into Con_Sea_1. Among the 601 Con_Seas with length less than 700 bp, Con_Sea_192, overlapping with *psaA-ycf3*, is the second highest intrageneric polymorphism region (PIC = 21.33). This region excludes the front section of *psaA-ycf3* (about 230 bp), which has low variation (PIC = 0.67), making it possible to be sequenced in a single Sanger reaction. In addition, *trnS-trnR* was divided into Con_Sea_26 and Con_Sea_27, both of which have relatively high intrageneric polymorphism, with PIC over 20 (Fig. 4c and Additional file 13: Table S4). In addition, several partial *ycf1* were identified as high intergeneric polymorphism regions (Fig. 4d and Additional file 13: Table S4).

### Evaluation of high variation regions

The PCR results showed that all the eight newly developed cp primers (Fig. 4c and d) were perfectly amplified in all 49 samples from 44 *Primulina* species. The levels of polymorphism of the eight markers developed from Con_Seas, compared with the four traditional loci developed from noncoding regions, were summarized in Additional file 15: Table S6. The regions amplified from cp primers of Con_Sea_1 (overlapping with *trnH-psbA*) had the highest PIC, which is consistent with the prediction by bioinformatics. The PIC ranking of 8 newly developed markers calculated by experimental evaluation are almost the same as that obtained from bioinformatic analysis, with the only difference in the exchange of the ranking of *rpoB-trnC* (listed as 3rd and 4th based on experiment and bioinformatic methods, respectively) and *rpl21-trnL* (listed as 4th and 3rd, respectively). Furthermore, the PIC values of 8 novel markers calculated by these two methods are highly correlated (*r* = 0.85, *p* = 0.0075) (Additional file 16: Fig. S2). In addition, consistent with our expectation, all the new markers were observed to have higher polymorphism than traditional ones with the exception for rpl32-trnL, which ranked in the 7th among the 12 loci tested. Interestingly, we found that the *rpl32-trnL* developed from Con_Seas method ranked higher in polymorphism than the locus developed from traditional noncoding regions (Additional file 15: Table S6). This result indicated that the new primer pairs seem to be a better choice for phylogenetic and population studies in *Primulina*.

### Sub-assembly of chloroplast genome from PE RAD-Seq (SACRing)

Compared to several popular restriction enzyme sites (REs), *EcoR I* was considered to be suitable for *Primulina* cp assembly from PE RAD-Seq, for its relative uniform distribution and modest number, which is between 107 and 117 in the 4 complete cp genomes obtained in this study (Fig. 3d). Therefore *EcoR I* was used in the library construction of 21 individuals, with the number of reads ranging from 145,876 and 797,450. These reads were isolated and used for further sub-assembly.

**Fig. 5:**
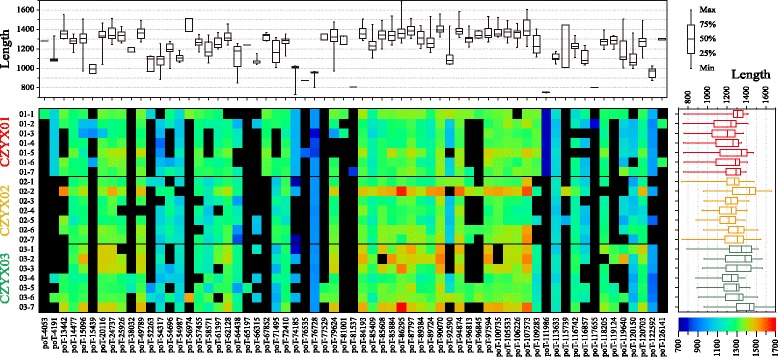
The heatmap of poTs among 21 individuals from three *Primulina* populations**.** poTs, short for paired & overlap tags, is a contig assembled from Tag1s (clustered from read1 of RAD-Seq) at forward and reverse directions of the same restriction enzyme site (RE) and their paired Tag2s (assembled from read2 of RAD-Seq). CpContigs are longer contigs further assembled from all kinds of RAD tags, including poTs. X axis of the heatmap showed the name of poTs, composed by “poT-” and a digit, while the digit represents the position of RE, around the middle position of this poT. The grids filled with different color represent the length of poTs at specific site and specific individual, with the bar shown in the bottom right corner. While black grids mean these poTs were not be assembled. The length distribution of 65 poTs and 21 individuals were shown at the top and the right of heat map, respectively, with the same bar shown at the top right corner

**Fig. 6:**
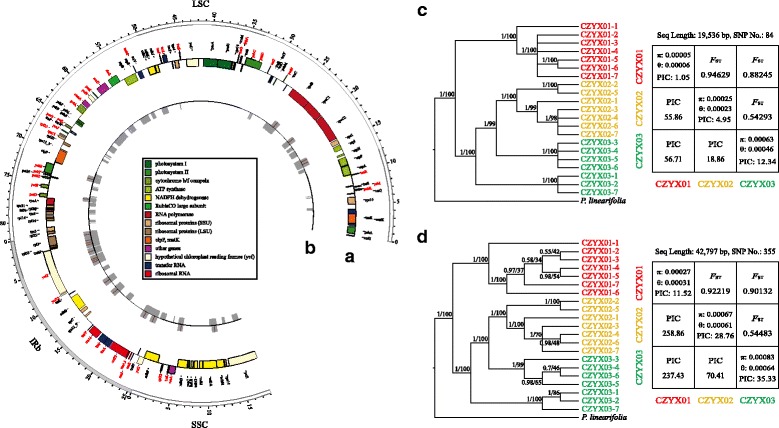
The distribution, polymorphic indexes of poTs and CpContigs in 21 individuals from three *Primulina* populations**.** poTs, short for paired & overlap tags, is a contig assembled from Tag1s (clustered from read1 of RAD-Seq) at forward and reverse directions of the same restriction enzyme site (RE) and their paired Tag2s (assembled from read2 of RAD-Seq). CpContigs are longer contigs further assembled from all kinds of RAD tags, including poTs. **a**, **b** The distribution of cp fragments (poTs) and CpContigs. The circle (**a**) showed the gene annotation of WHY01 (*Primulina eburnea*). Genes shown inside and outside of the circle are transcribed clockwise and counterclockwise, respectively, and their gene names are marked as black and red, respectively. Genes belonging to different groups are marked with different color, with the bar shown in the center. The outside and inside of circle (**b**) showed the distribution of cp fragments (poTs) and CpContigs, respectively. And brown line in the outside of this circle represented the position of REs. **c**, **d** The Bayesian / Maximum Likelihood phylogenetic tree and population genetic indexes based on concatenate sequences of poTs (**c**) and CpContigs (**d**). Posterior probabilities >0.5 in BI analysis and bootstrap values >50% in ML analysis are indicated on the left and right of slash respectively

The average number of RAD tags in CZYX01 (*P. eburnea*) is 154, slightly higher than that in CZYX02 (*P. yongxingensis*) (142) and CZYX03 (*P. juliae*) (144) (Table 2). The percentage of pTs (paired tags, mixtures of two Tag1s at forward and reverse directions of the same restriction enzyme site), oTs (overlap tags, mixtures of Tag1 and its paired Tag2, which was sub-assembled from read2, according to the overlap) and poTs (paired & overlap tags, mixtures of two paired RAD Tag1s and both of their paired Tag2s) among the three populations is 80–84%, 80–81%, and 59–61%, respectively (Table 2). The length of poT ranged from 734 to 1692 bp, with the mean value of 1257 bp. Over 93.2% of poTs have a length over 1000 bp (Fig. 5). The comparative analysis showed that the 20 most conserved poTs were obtained in all the individuals, and they have relative consistent and longer length, 90% of which have an average length over 1240 bp (Fig. 5). And the consistent sequences of 20 poTs were further assembled into 14 cp fragments, with the total length of 19,536 bp, taking over 15% of the cp genomes (Fig. 6a and b). The Maximum Likelihood and Bayesian phylogenetic tree showed that these 21 individuals could be divided into four groups, with CZYX03 split into two groups (Fig. 6c). Meanwhile, 84 SNP sites were identified from 14 cp fragments among 21 individuals. CZYX03 showed the highest intra-population variation, followed by CZYX02 and CZYX01. The inter-population genetic differentiation between CZYX02 and CZYX03 is significantly lower than that of others (Fig. 6c).

**Table 2:** The basic information of 21 *Primulina* RAD-Seq data related to chloroplast genomes

Species	ID	Reads No.^a^	Quality Q30^b^	RAD tags No.^c^	Paired tags No. - ratio (%)^d^	Overlap tags No. - ratio (%)^e^	Paired & Overlap tags No. - ratio (%)^f^	Cpcontigs^g^		
								No.	Length	Ratio (%)^h^
*P. eburnea*	CZYX01–1	320,451	96.0; 97.2	164	73 (×2) - 89	132 - 80	50 (×2) - 61	62	69,862	55.1
	CZYX01–2	239,167	96.3; 95.7	151	64 (×2) - 85	130 - 86	52 (×2) - 69	62	65,591	51.7
	CZYX01–3	426,610	94.7; 96.1	151	61 (×2) - 81	121 - 80	44 (×2) - 58	75	63,584	50.1
	CZYX01–4	474,623	95.4; 96.4	153	63 (×2) - 82	119 - 78	43 (×2) - 56	76	62,588	49.3
	CZYX01–5	797,450	95.4; 96.6	148	59 (×2) - 80	115 - 78	40 (×2) - 54	79	65,103	51.3
	CZYX01–6	427,701	96.4; 95.6	154	64 (×2) - 83	126 - 82	48 (×2) - 62	70	67,315	53.0
	CZYX01–7	404,182	96.4; 96.3	156	67 (×2) - 86	129 - 83	50 (×2) - 64	65	66,895	52.7
	CZYX01^i^	441,455	95.8; 96.3	154	64 (×2) - 84	125 - 81	47 (×2) - 61	70	65,848	51.9
*P. danxiaensis*	CZYX02–1	278,229	96.2; 97.4	145	58 (×2) - 80	117 - 81	42 (×2) - 58	68	62,883	49.6
	CZYX02–2	343,741	96.0; 96.1	145	61 (×2) - 84	111 - 77	44 (×2) - 61	69	65,103	51.3
	CZYX02–3	228,868	96.2; 95.5	137	52 (×2) - 76	109 - 80	38 (×2) - 55	72	58,660	46.2
	CZYX02–4	230,025	96.2; 95.4	138	53 (×2) - 77	110 - 80	38 (×2) - 55	72	58,696	46.3
	CZYX02–5	211,979	96.3; 95.6	140	56 (×2) - 80	115 - 82	46 (×2) - 66	71	61,728	48.6
	CZYX02–6	227,541	96.3; 96.5	145	59 (×2) - 81	117 - 81	44 (×2) - 61	69	61,609	48.6
	CZYX02–7	277,044	96.3; 96.5	143	57 (×2) - 80	111 - 78	42 (×2) - 59	75	63,284	49.9
	CZYX02^j^	256,775	96.2; 96.1	142	57 (×2) - 80	113 - 80	43 (×2) - 59	71	61,709	48.6
*P. juliae*	CZYX03–1	208,356	95.9; 96.0	141	60 (×2) - 85	114 - 81	42 (×2) - 60	61	63,457	50.0
	CZYX03–2	315,085	95.9; 95.9	142	60 (×2) - 85	109 - 77	40 (×2) - 56	65	64,130	50.5
	CZYX03–3	172,588	96.1; 96.7	139	54 (×2) - 78	110 - 79	39 (×2) - 56	71	65,415	51.6
	CZYX03–4	145,876	96.3; 96.1	139	54 (×2) - 78	116 - 83	45 (×2) - 65	68	61,485	48.5
	CZYX03–5	694,697	96.3; 97.4	155	65 (×2) - 84	126 - 81	40 (×2) - 52	69	65,392	51.5
	CZYX03–6	333,471	96.1; 97.1	154	63 (×2) - 82	129 - 84	52 (×2) - 68	67	69,166	54.5
	CZYX03–7	381,048	95.9; 96.0	140	58 (×2) - 83	112 - 80	41 (×2) - 59	61	63,551	50.1
	CZYX03^k^	321,589	96.1; 96.5	144	59 (×2) - 82	117 - 81	43 (×2) - 59	65	64,647	51.0

^a^Reads No. was counted from the Reads which was mapped into the reference cp genome *P.eburnea* (WHY01), instead of whole sequencing data.

^b^Quality Q30 was counted from Read1 and Read2 respectively.

^c^RAD tags No.: The number of Tag1s, clustered from read1 of RAD-Seq.

^d^Paired tags No. & ratio: The number of pTs (paired tags) and the value of pTs No. / RAD tags No., where pT was mixed from two Tag1s at forward and reverse directions of the same RE (restriction enzyme site).

^e^Overlap tag No. & ratio: The number of oTs (overlap tags) and the value of oTs No. / RAD tags No., where oT was mixed from Tag1 and its paired Tag2 (assembly of read2) according to the overlap.

^f^Paired & Overlap tags No. & ratio: The number of poTs (paired & overlap tags) and the value of poTs No. / RAD tags No., where poT was mixed from paired tags (two Tag1s) and their paired Tag2s (two Tag2s). Both of two Tag1 have overlap regions with paired Tag2s.

^g^Cpcontigs: it is a longer sequences without unknown nucleotides, and it was assembled from all the kinds of tags, including pTs, oTs, poTs and other types, according to the their position and overlap information.

^h^Ratio: it was counted as following: The length of Cpcontigs / the length of reference cp genome of *P.eburnea* (WHY01), while the cp genome length here is 126,890, excluding the length of IRa.

^i^CZYX01, ^j^CZYX02, ^k^CZYX03: they represent the index in the species level, which was calculated by the average level of 7 individuals from the same species

The average number of CpContigs in CZYX01, CZYX02 and CZYX03 is 70, 71 and 65, occupying around 52%, 49% and 51% of the entire cp genome, respectively (Table 2). Furthermore, 62 consistent CpContigs, which were distributed equably across whole cp genome, were isolated, with the total length of 42,797 bp, accounting for over one third of the cp genome (Fig. 6a and b). A total of 355 SNP were identified from the CpContig, over four folds of that from the poT dataset (84 SNPs). Nevertheless, the results of population genetic analysis of the cp concatenated sequences from consistent CpContigs are similar to that from consistent poTs (Fig. 6c and d). These results indicated that around half of *Primulina* cp genome could be directly assembled from RAD-Seq data through our SACRing pipeline. Both poTs and CpContigs could provide enough cp information and variant SNP sites for phylogenetic and population genetic studies.

## Discussion

### Lineage-specific high variable regions

With the rapid development and wide application of NGS technology, it has become much easier to obtain complete chloroplast genomes, as evidenced by the dramatic increase in the number that are publically available (http://www.ncbi.nlm.nih.gov/genome/browse/). Recent comparative plastid genomic studies reveal a pattern of lineage-specific high variable regions in different lineages [3, 7–9]. For example, the most variable regions identified in the genus *Pyrus* are *ndhC-trnV*, *trnR-atpA*, *ndhF-rpl32*, *psbM-trnD*, and *trnQ-rps16*, while only two (*ndhF-rpl32* and *trnK-rps16*) were consistently found among the Shaw et al., [6] top-ranked 30 cpDNA regions [9]. In this study, the top-9 ranked high variable regions in *Primulina* overlap with *trnH-psbA*, *ndhF-rpl32*, *trnC-petN*, *psaA-ycf3*, *rpl32-trnL*, *trnS-trnR*, *psbI-trnS-trnR*, *rpoB-trnC* and *ndhH-rps15-ycf1* (Fig. 4c). Of them, only five are listed among the top-ranked 34 cpDNA regions by Shaw et al., [6]. In contrast, the most variable regions identified between the genera *Primulina* and *Boea* overlap with *rps16-trnQ*, *atpH-atpI*, *ndhF-rpl32*, *rpoB-trnC* and *trnK-rps16* (Fig. 4d), all of which are listed top 14th highest variable regions by Shaw et al., [6]. Our experimental tests confirmed that the new markers developed from *Primulina* cp genomes can provide higher polymorphism than the traditional cp primers developed from noncoding regions of distantly related angiosperms. Although listed in top-ranked variable regions, these traditional markers show only moderate variation in *Primulina* cp genomes (Additional file 15: Table S6). These results imply that the cp markers developed from the three *Primulina* cp genomes are more suitable in phylogenetic and population studies of *Primulina* than the traditional and universal cp markers. On the other hand, the makers developed from high variable regions between *Primulina* and *Boea* can be used in the higher level phylogeny analysis in the Gesneriaceae family.

### A novel strategy for determination of high variation regions: From noncoding regions to Con_Seas regions

Chloroplast DNA markers are usually developed from noncoding regions with high variation [5, 6]. Here we proposed an improved method to develop cp primers from truly high variation regions (i.e., Con_Seas, the regions between two adjacent conserved regions) across whole cp genomes, instead of noncoding regions. These two methods were further compared systematically and globally. Our newly proposed method has several advantages. First, continuous conserved regions and synthetic coding regions are not matched perfectly, and partial Con_Islands (the conserved regions in cross-genus consensus genome sequences) exist in noncoding regions (Fig. 3a and b). For example, the length of *trnS-trnR* is too long to be sequenced entirely with two sequencing reactions although this region has both the highest intrageneric and intergeneric PIC (Fig. 4a and b). The *trnS-trnR* region cannot be used for developing markers with the traditional strategy. Since there is a 76 bp Con_Island in the middle of this region (Fig. 3a and b), the *trnS-trnR* region was divided into two high variation regions according to our Con_Seas method, both of which are suitable for development of cp primers. Second, long functional genes usually have many mutation hotspot regions. For example, the 5.5-kb long *ycf1* gene has nine Con_Seas but no introns, and several Con_Seas have high mutational hotspots, especially in intergeneric polymorphism (Fig. 3a, b and c). These regions were ignored in previous marker development; however, several highly variable regions were identified in our method (Fig. 4c and d), and the polymorphism of one locus has been verified experimentally (Additional file 15: Table S6). Third and similarly, the boundaries of coding regions of functional genes (or even the entire coding regions) are not always conserved enough to develop perfect cp primers, especially in SSC region (Fig. 3a and b). Therefore, it is difficult for developing suitable markers using traditional methods, while our method could avoid this problem to a remarkable extent.

In this study, 18 Con_Seas have both intrageneric and intergeneric PICs higher than that of Con_Sea_124 (*trnE-trnT*), which is 9th most variable in our experimental evaluation (Additional file 13: Table S4 and Additional file 15: Table S6). The highly variable nature of these loci will greatly assist phylogenetic studies in *Primulina*. We believe that this strategy for determination of high variation regions based on Con_Seas would provide a reference for development of high polymorphic cp markers of other plants with whole cp genomes. Alignment of two plastomes could be supported by our software “Con_Sea_Identification_and_PIC_Calculation”, but the result may have a poor positive correlation with the true variability across the taxon of interest if only two taxa are used. We recommended that at least three cp genomes be available when using it. It was noteworthy that this strategy and software needs to be continually optimized by any user, to determine setting how to select the best parameter of minimum length of Con_Island. The adjacent Con_Seas with relative short length could be joined up as a new region, in order to provide the most variable characters in one or two Sanger sequencing reactions.

### A novel strategy to generate cp sequences for phylogenetic studies: From sanger sequencing to PE RAD-Seq

RAD-Seq is increasingly used in population genetic and phylogenetic studies, due to the rapidly decreasing cost of sequencing [47]. However, only nuclear genetic information is typically extracted and analyzed. In this study, we have developed a novel strategy to obtain large amount of cp variable characters directly from RAD-Seq. Using our pipeline SACRing, around 44 cp sequences with the average length of 1260 bp, and 64-kb-long CpContigs (sub-super-marker, half of whole cp genome) were obtained from RAD-Seq of the 21 *Primulina* individuals, which we used for population genetic analyses (Table 2 and Fig. 5). We believe that having publically available tools to handle cp data in RAD-Seq datasets could allow those working with nuclear RAD-Seq datasets to enrich them with cp genome data at no further cost, greatly increasing their value.

Complete cp genome sequences were recently proposed as super marker for DNA barcoding of plants, which could greatly improve resolution [20, 48]. However, recent studies revealed that such a super-marker may not substantially improve discrimination of clades that recently diverged or that have complex patterns of hybridization [49–51]. Furthermore, it is still not easy for researchers without programming background to isolate and assemble cp genomes from NGS data. In addition, many experimental aids or manual corrections are required in the steps of gap closing and annotation. Therefore, complete cp genomes for dozens or hundreds of individuals is still impractical in most studies. Using the pipeline SACRing, a sub-super-marker (CpContigs), comprising *c*. 50% of entire cp genomes, could be easily obtained for hundreds of individuals in a fully automated approach and in a relative short amount of time. Compared with super-markers (i.e., entire cp genomes, which was assembled from genome survey sequencing), our sub-super-marker (CpContigs) or even poTs could provide sufficient variable characters but with lower cost. RAD-Seq with specific restriction enzyme site could obtain enough raw NGS data for cp genome assembly at a much lower sequencing throughput, *c*. 5–10% of that of genome survey sequencing.

On the other hand, some previous studies have used resequencing (mapping the NGS reads to the reference cp genomes directly) to call the SNPs of cp DNA [52]. However, such direct mapping approaches lead to errors of intra-individual polymorphism because of DNA transfer of cp sequences into the nuclear or mitochondrial genomes or the both [53]. In our strategy, the assembly of poTs or CpContigs seem to be accurate due to the high abundance of chloroplast genomes, which are 1–2 orders of magnitude of mitochondrial genome, and 2–4 orders of magnitude of nuclear genome in a single leaf cell [52]. Because variable characters were identified based on cluster results of poTs or CpContigs, candidate assembly errors could be identified. Therefore, our strategy could minimize spurious intra-individual polymorphism.

To the best of our knowledge, this study is the first to develop the sub-assembly of poTs and CpContigs (sub-super-marker, *c*. 50% of entire cp genomes) from PE RAD-Seq, and the SACRing was the first pipeline to bridge the relationship between RAD-Seq and cp genomes. The pipeline can be download from https://github.com/scbgfengchao/, where it will be subjected to continual improved and updated. This strategy expands the application of RAD-Seq, and would be practice for large-scale application of poTs or sub-super-marker in phylogenetic and population genetic studies.

We recommend that in the cases where only dozens of variable sites are required and where at least 3 cp genomes of related species are available, the first strategy "from noncoding regions to Con_Seas regions" will be more effective. Otherwise, our second strategy is recommended, particularly when only a single cp genome of closely related species is available. We also recommend the second method when the aim is to supplement a population genetics study using RAD-seq at nuclear loci with cp loci.

## Conclusions

In this study, three complete *Primulina* chloroplast genomes were assembled from genome survey data. Combined with the cp genome of *Boea hygrometrica*, several lineage-specific highly variable cp markers were developed from the true high variation regions (Con_Seas) across whole cp genomes using the software “Con_Sea_Identification_and_PIC_Calculation”. This approach provided higher polymorphism than traditional cp primers, which was confirmed by experimental evaluation results. The newly developed markers will promote phylogenetic and population genetic studies in *Primulina* and other genera of the family Gesneriaceae.

We also wrote a novel Bash script, SACRing, which uses RAD-Seq data to directly assemble cp fragments (poTs) and sub-super-marker (CpContigs), representing around half of the completed cp genome (in the case of 21 individuals from three *Primulina* populations). The conserved poTs or CpContigs could be further applied in the studies of population genetic analysis and phylogenetics. Our method fundamentally alters traditional approaches, which have been deeply dependent on large amounts of Sanger sequencing based on cp primers. These two novel strategies proposed in our study may provide a reference to similar research in other non-model species fascinated by next generation sequencing.

## Additional files

Additional file 1: Table S1. The list of 49 individuals from 44 *Primulina* species used for evaluation of the polymorphism of chloroplast markers and GenBank accession id of 12 cp sequences for each individual (XLSX 16 kb)

Additional file 2: File S1. The script of QC (quality control of Illumina PE reads) (ZIP 4 kb)

Additional file 3: File S2. The script of Correspondence_between_each_seq_and_consensus (ZIP 6 kb)

Additional file 4: File S3. The script of Con_Sea_Identification_and_PIC_Calcalation (ZIP 182 kb)

Additional file 5: File S4. The script of PIC_calculation (Calculation of SNP No., InDel No. and PIC values between two sequences after alignment) (ZIP 14 kb)

Additional file 6: File S5. The script of SACRing (Sub-Assembly of Chloroplast genome from PE RAD-seq) (ZIP 31264 kb)

Additional file 7: File S6. The script of RAD_QC_pe (Quality Control of PE RAD-seq) (ZIP 5 kb)

Additional file 8: Figure S1. The mapping depth and coverage of three Primulina chloroplast genomes shown in Integrative Genomics Viewer, *P. eburnea* (a), *P. huaijiensis* (b), *P. linearifolia* (c) (PDF 819 kb)

Additional file 9: File S7. The gff3 files of *P. eburnea*, *P. huaijiensis*, *P. linearifolia* and *B. hygrometrica (ZIP 22 kb)*

Additional file 10: Table S2. Feature of function genes and syntenic coding/noncoding loci of four chloroplast genomes and consensus sequences (XLSX 406 kb)

Additional file 11: Table S3. Intrageneric and Intergeneric polymorphism of each syntenic coding loci and syntenic noncoding loci (XLSX 41 kb)

Additional file 12: File S8. The cluster result of four chloroplast genomes of *P. eburnea*, *P. huaijiensis*, *P. linearifolia* and *B. hygrometrica (FAS 609 kb)*

Additional file 13: Table S4. Intrageneric and Intergeneric polymorphism of each Con_Sea region (XLSX 73 kb)

Additional file 14: Table S5. Intrageneric and Intergeneric polymorphism of each window region (XLSX 583 kb)

Additional file 15: Table S6. The bioinformatic analysis and experimental evaluation of eight newly developed chloroplast markers and four traditional ones (XLSX 12 kb)

Additional file 16: Figure S2. The PIC values of eight newly developed chloroplast markers calculated by bioinformatic analysis and experimental evaluation (PDF 222 kb)

## Abbreviations

Con_Islands: regions containing over 50 continuous conserved sites in the cross-genus consensus genome sequences
Con_Seas: the regions between two adjacent Con_Island
cp: chloroplast
IR: inverted repeat
ITS: nuclear ribosomal internal transcribed spacer
LSC: large single copy
NGS: next-generation sequencing
oTs: overlap tags
PE: paired-end
PIC: potentially informative characters
poTs: paired & overlap tags
pTs: paired tags
RAD-Seq: restriction-site associated DNA sequencing
SACRing: Sub-assembly of chloroplast from PE RAD-Seq
SSC: small single copy
