# Supplementary material for: The complete chloroplast genome of Primulina and two novel strategies for development of high polymorphic loci for population genetic and phylogenetic studies
Source: BMC Evol Biol. 2017 Nov 7;17:224. doi: 10.1186/s12862-017-1067-z (PMC5678776; doi:10.1186/s12862-017-1067-z)
Supplement: Supplementary file 6 — The script of SACRing (Sub-Assembly of Chloroplast genome from PE RAD-seq) (ZIP 31264 kb) [file 12862_2017_1067_MOESM6_ESM.zip › SACRing-master/version_1.1/pipeline.pdf]

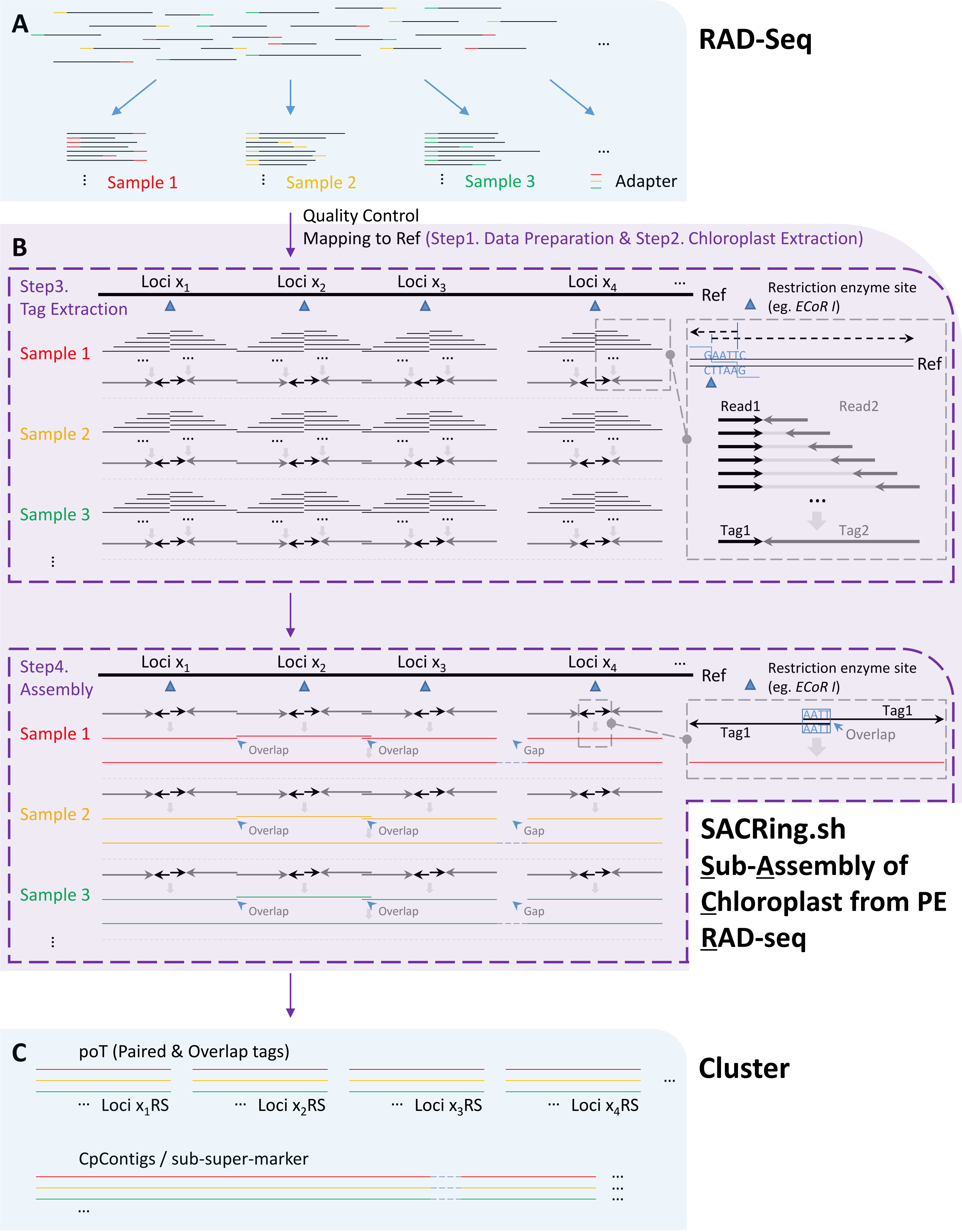

A
RAD-Seq
Sample 1
Sample 2
Sample 3
Adapter
Quality Control
Mapping to Ref (Step1. Data Preparation & Step2. Chloroplast Extraction)
B
Step3.
Tag Extraction
Loci x_1
Loci x_2
Loci x_3
Loci x_4
Ref
Restriction enzyme site
(eg. *ECoR I*)
GAATTC
CTTAAG
Read1
Read2
Tag1
Tag2
Step4.
Assembly
Overlap
Gap
AATT
SACRing.sh
Sub-Assembly of Chloroplast from PE RAD-seq
C
poT (Paired & Overlap tags)
Loci x_1RS
Loci x_2RS
Loci x_3RS
Loci x_4RS
Cluster
CpContigs / sub-super-marker
