# Supplementary figures and images for: The complete chloroplast genome of Primulina and two novel strategies for development of high polymorphic loci for population genetic and phylogenetic studies
Source: BMC Evol Biol. 2017 Nov 7;17:224. doi: 10.1186/s12862-017-1067-z (PMC5678776; doi:10.1186/s12862-017-1067-z)

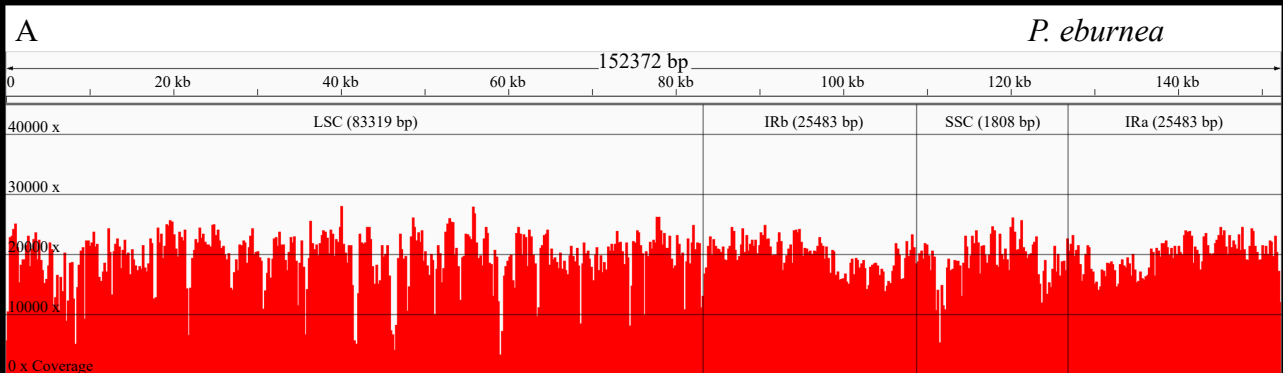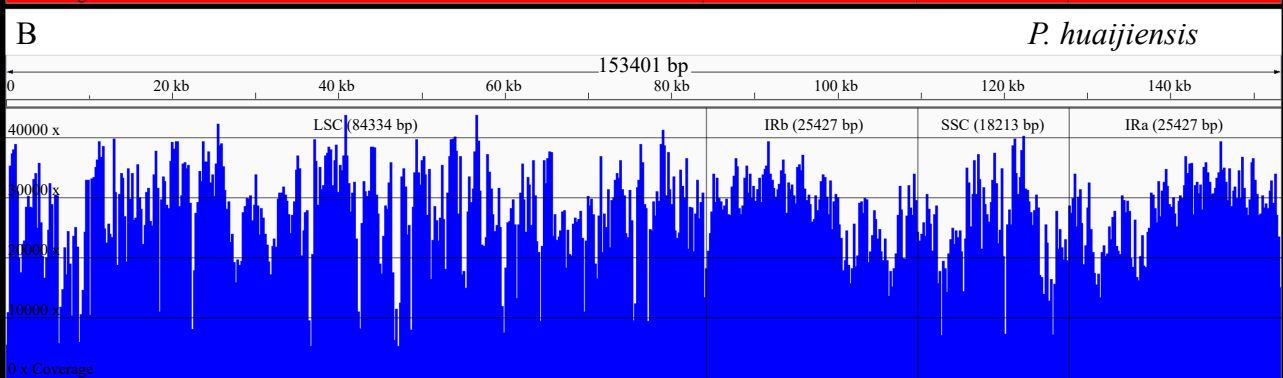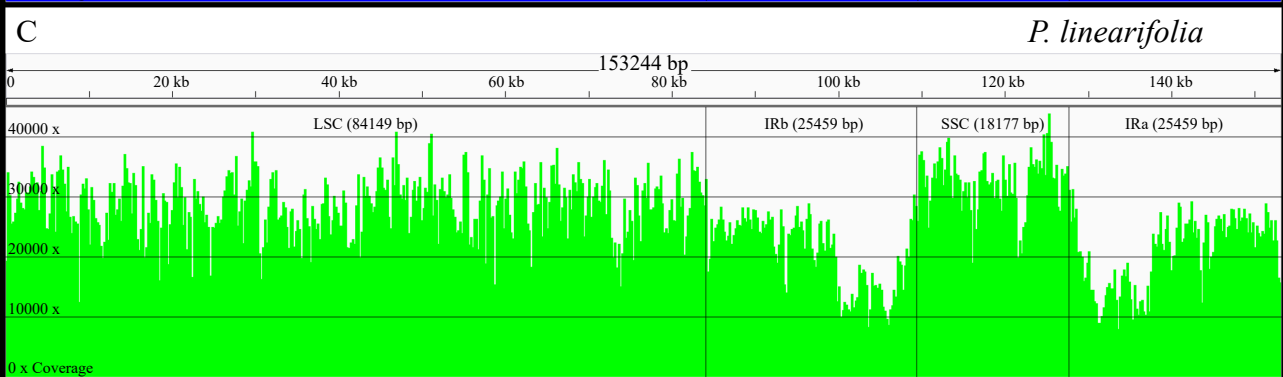

Supplement: Supplementary file 8 — The mapping depth and coverage of three Primulina chloroplast genomes shown in Integrative Genomics Viewer, P. eburnea (a), P. huaijiensis (b), P. linearifolia (c) (PDF 819 kb) [file 12862_2017_1067_MOESM8_ESM.pdf]

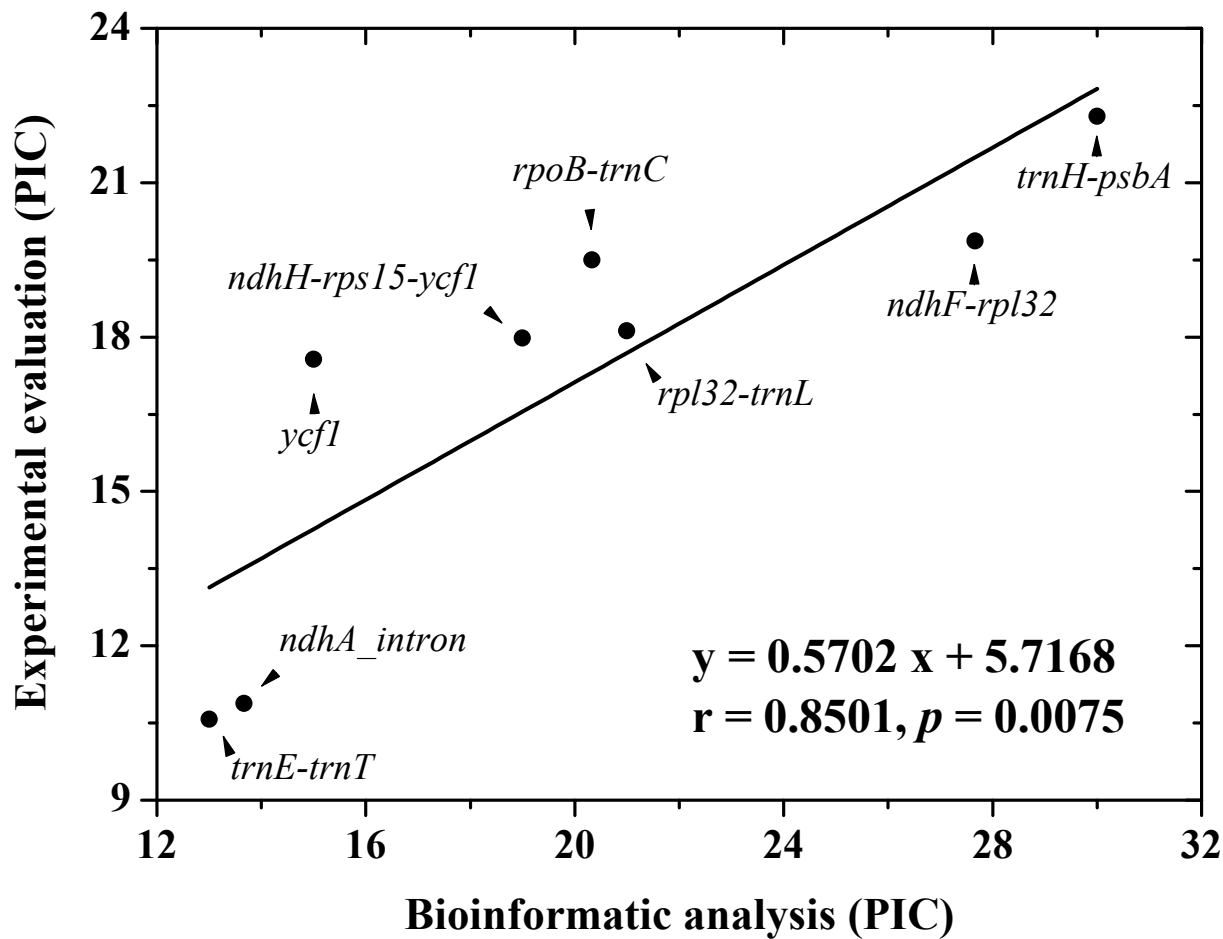

Supplement: Supplementary file 16 — The PIC values of eight newly developed chloroplast markers calculated by bioinformatic analysis and experimental evaluation (PDF 222 kb) [file 12862_2017_1067_MOESM16_ESM.pdf]
